# Blocked conversion of *Lactobacillus johnsonii* derived acetate to butyrate mediates copper-induced epithelial barrier damage in a pig model

**DOI:** 10.1186/s40168-023-01655-2

**Published:** 2023-09-30

**Authors:** Yang Wen, Luqing Yang, Zhenyu Wang, Xiaoyi Liu, Meng Gao, Yunhui Zhang, Junjun Wang, Pingli He

**Affiliations:** 1 State Key Laboratory of Animal Nutrition, Frontiers Science Center for Molecular Design Breeding (MOE), China Agricultural University, No. 2 Yuanmingyuan West Road, Beijing, 100193 China; 2 State Key Laboratory of Farm Animal Biotech Breeding, College of Biological Sciences, China Agricultural University, Beijing, 100193 China; 3 College of Environmental Science and Engineering, Tongji University, Shanghai, 200092 China

**Keywords:** *Lactobacillus johnsonii*, Colonic barrier, Short-chain fatty acid, Microbiota, Copper

## Abstract

**Background:**

High-copper diets have been widely used to promote growth performance of pigs, but excess copper supplementation can also produce negative effects on ecosystem stability and organism health. High-copper supplementation can damage the intestinal barrier and disturb the gut microbiome community. However, the specific relationship between high-copper-induced intestinal damage and gut microbiota or its metabolites is unclear.

**Objective:**

Using fecal microbiota transplantation and metagenomic sequencing, responses of colonic microbiota to a high-copper diet was profiled. In addition, via comparison of specific bacteria and its metabolites rescue, we investigated a network of bacteria-metabolite interactions involving conversion of specific metabolites as a key mechanism linked to copper-induced damage of the colon.

**Results:**

High copper induced colonic damage, *Lactobacillus* extinction, and reduction of SCFA (acetate and butyrate) concentrations in pigs. LefSe analysis and q-PCR results confirmed the extinction of *L. johnsonii*. In addition, transplanting copper-rich fecal microbiota to ABX mice reproduced the gut characteristics of the pig donors. Then, *L. johnsonii* rescue could restore decreased SCFAs (mainly acetate and butyrate) and colonic barrier damage including thinner mucus layer, reduced colon length, and tight junction protein dysfunction. Given that acetate and butyrate concentrations exhibited a positive correlation with *L. johnsonii* abundance, we investigated how *L. johnsonii* exerted its effects by supplementing acetate and butyrate. *L. johnsonii* and butyrate administration but not acetate could correct the damaged colonic barrier. Acetate administration had no effects on butyrate concentration, indicating blocked conversion from acetate to butyrate. Furthermore, *L. johnsonii* rescue enriched a series of genera with butyrate-producing ability, mainly *Lachnospiraceae NK4A136 group*.

**Conclusions:**

For the first time, we reveal the microbiota-mediated mechanism of high-copper-induced colonic damage in piglets. A high-copper diet can induce extinction of *L. johnsonii* which leads to colonic barrier damage and loss of SCFA production. Re-establishment of *L. johnsonii* normalizes the SCFA-producing pathway and restores colonic barrier function. Mechanistically, *Lachnospiraceae NK4A136 group* mediated conversion of acetate produced by *L. johnsonii* to butyrate is indispensable in the protection of colonic barrier function. Collectively, these findings provide a feasible mitigation strategy for gut damage caused by high-copper diets.

Video Abstract

**Supplementary Information:**

The online version contains supplementary material available at 10.1186/s40168-023-01655-2.

## Introduction

Copper is an essential element for animals, which has been used widely as a feed additive to promote growth performance and reduce frequency of diarrhea in pigs [1, 2]. On the other hand, excess intake of copper also has negative effects on animals and humans, such as co-evolution of metal and antibiotic resistance, and copper contamination of natural environment [3–5]. Recently, researchers have reported that high-copper diet can destroy structure and function of the gut epithelial barrier in piglets leading to increased gut epithelial leakage [6]. However, the factors mediating high-copper-induced intestinal barrier damage are still unclear.

The intestine is the home of a microbial community that includes more than 10^14^ microorganisms [7]. Gut microbiota is crucial in serving as “defenders” to protect gut health against invading xenobiotics. The intestine is the main interface between the host and dietary copper exposure. Previous researcher revealed that copper supplementation may interact directly with bacterial flora in the intestines causing death of commensal microbiota and increased proliferation of pathogenic bacteria [8]. Specifically, high dietary copper decreased Firmicutes (especially for *Lactobacillus*) in the pig colon. *Lactobacillus* is an important short-chain fatty acid (SCFA) producer [9]. Furthermore, feeding piglets a high-copper diet decreased relative abundance of butyrate-producing bacteria, such as *Lachnospiraceae*, *Acidaminococcus*, *Coprococcus*, and *Roseburia* [8]. Liao et al. found increased levels of pathogenic bacteria, such as *Streptococcus* and *Enterobacteriaceae*, could aggravate colonic damage during copper exposure [6].

The gut microbiome is fueled by dietary macronutrients to produce bioactive compounds consisting of bile acids, short-chain fatty acids, and indoles [10]. These microbiota-derived bioactive substances act as agents of microbe-host communication, which is essential for maintaining normal host physiology. Microbial conversions of undigested fibers to SCFA are mediated by specific gut bacteria. Pathways for acetate production are distributed widely among bacterial groups like *Lactobacillus*, while butyrate production pathways appear more conserved and species specific [11]. A relatively small number of gut bacteria, such as *Lachnospiraceae*, *Roseburia*, and *Clostridium butyricum*, can convert acetate to butyrate [12]. Butyrate promotes intestinal barrier function in various animal models because it serves as a substrate of beta-oxidation [13]. Gut dysbiosis caused by high-copper feeding of pigs decreases concentrations of SCFA [14]. Based on the negative effects of high copper on microorganisms, it is still not clear whether changes in microbial metabolites are potential contributors to colonic barrier damage.

In the present study, we aim to investigate the mechanistic connections among copper-induced colonic damage and the microbiota and microbial metabolites. Using fecal microbiota transplantation and shotgun metagenomic sequencing, copper-induced gut dysbiosis characterized by *L. johnsonii* extinction was found inducing colonic barrier damage. *L. johnsonii* rescue and SCFA administration experiments demonstrated that the resupply of acetate and the restoration of defective butyrate synthesis were the mechanisms by which *L. johnsonii* exerted its protective effect.

## Materials and methods

### Animals and sample collection

All experiments were performed in accordance with principles and guidelines for use of laboratory animals of China Agricultural University and approved by the Institutional Animal Care and Use Committee of China Agricultural University.

#### Weaned piglet experiment

This experiment was conducted at the swine research unit of China Agricultural University (Chengde, China). The selected piglets were 28 days old (weaned at day 21 and fed creep feed after weaning for 7 days) with their initial body weight (BW) of 8.32 ± 0.51 kg. A total of 72 Duroc × (Landrace × Yorkshire) crossbred piglets were allotted randomly into two treatments by their sex (half male and half female) and weighted. Each treatment contained six replicate pens with six piglets per pen. This experiment lasted for 42 days and included two periods, (1) copper treatment (0 ~ 28 days): copper-free diet (with no supplemental copper) and copper-rich diet (with 120 mg Cu/kg supplied by copper sulfate); (2) recovery (29 ~ 42 days): when copper-free diet was fed to all pigs. Feed samples were collected for determination of copper contents. All diets were formulated to meet or exceed nutrient requirements for growing pigs (NRC 2012) as listed in Additional file 1: Table S1. No antibiotics or other animal health products were used. Water and diets were provided ad libitum for piglets. The relative humidity and temperature of the piglet house were set at 60–65% and 25–28 °C, respectively. Body weight was measured on 0, 14, 28, and 42 days of the experiment. Diarrhea score was estimated visually every 3 days according to Lin et al*.* [15]. Fresh feces were collected by swab on days 14, 28, and 42 for FMT, and analysis of gut microbiota and metabolites. One pig per pen was placed in a separate pen without access to feed and water for 12 h on day 28. Selected pigs (*n* = 12) were euthanized by electrical stunning and exsanguination. Cotton rope was first used to prevent digesta from moving between intestinal segments during sampling. Fresh digesta within the jejunum and the proximal colon were collected. All samples were frozen immediately in liquid nitrogen and stored at − 80 °C. Ten-millimeter segments of proximal colon were flushed with PBS and fixed in 4% paraformaldehyde for histological analysis. Remaining tissues were frozen in liquid nitrogen for future analysis.

#### Mouse experiments

To investigate the mechanisms of copper-induced colon damage with respect to involvement of the microbiota and microbial metabolites, additional two experiments were conducted using 6- to 7-week-old SPF male C57BL/6 J (SPF Biotechnology Co., Ltd., China) mice. All mice were housed in a standard SPF facility of China Agricultural University with controlled temperature (22 °C) and light conditions (14 h light, 10 h darkness; lights on at 07:00 a.m.). Experimental mice were firstly treated by antibiotic to clean bacteria.

#### Experiment for bacterial rescue

A total of 36 mice were divided randomly into three groups as follows: (1) CF-FMT group: Fecal bacterial suspension collected on day 28 from pigs fed copper-free diet; (2) CR-FMT group: Fecal bacterial suspension collected on day 28 from pigs fed copper-rich diet; and (3) CR-Lj group: CR-FMT with 1 × 10^9^ *L. johnsonii* YXY13 colony forming units (CFU) per mouse.

#### Experiment for SCFAs validation

A total of 60 mice were divided randomly into five groups as follows: (1) CF-FMT group: Fecal bacterial suspension collected on day 28 from pigs fed copper-free diet; (2) CR-FMT group: Fecal bacterial suspension collected on day 28 from pigs fed copper-rich diet; (3) CR-Lj group: CR-FMT with 1 × 10^9^ *L. johnsonii* YXY13 CFU per mouse; (4) CR-A group: CR-FMT with 200 mM sodium acetate (S2889, Sigma Aldrich, USA) solution in drinking water; (5) CR-B group: CR-FMT with 100 mM sodium butyrate (S303410, Sigma Aldrich, USA) solution in drinking water. All mice were gavage daily with the corresponding treatment solution for 14 days. These two experiments used the same sampling protocol. Body weight was measured on days 14 and 28. All mice were sacrificed via anesthesia at the end of the experiment. The colon length was first measured. A 5-mm segment of the proximal colon was flushed with PBS and then fixed in 4% paraformaldehyde, and then the remaining tissues were frozen in liquid nitrogen for future analysis. Fresh colonic digesta were collected in three 2-mL tubes and immediately frozen in liquid nitrogen, and stored at − 80 °C for further analysis.

#### Cell culture and treatments

To explore and screen for strains with potential protective functions against the intestinal barrier, another experiment was conducted using cell line HT-29 (provided by Dr. Wang of China Agricultural University, Beijing). Cells were routinely cultured in DMEM medium supplemented with 10% (v/v) FBS (10,099,141, Gibco, USA) and 10 mM HEPES buffer (15,630,106, Gibco, USA) and maintained at 37 °C with 5% CO_2_ and 95% air atmosphere with 90% humidity. No antibiotics were used in the medium. To form a monolayer, HT-29 cells were seeded onto 6.5-mm-diameter Transwell polyester inserts (3 μm pore size) at a density of 1 × 10^5^ cells per insert and cultured for 8 to 9 days until differentiation. Cells were then incubated for 20 h with 1 × 10^8^ CFU/mL of different *L. johnsonii* to identify the strains with the potential to promote intestinal barrier via measurement of transepithelial electrical resistance (TEER, MERS00002, Millipore, USA). Three selected strains based on TEER were subjected to subsequent experiment. HT-29 cells were seeded in standard 12-well plates at a density of 1 × 10^6^ cells per well and maintained until fluency. Then HT-29 cells were treated for 6 h in serum-free medium. Experimental treatments were as follows: (1) CON group: PBS; (2) CS group: 200 mM copper sulfate; (3 to 5) YXY11/12/13 groups: 200 mM copper sulfate and 1 × 10^8^ CFU/mL *L. johnsonii* YXY11/12/13.

### Isolation and culture of *L. johnsonii*

To isolate *L. johnsonii*, fecal samples from day 28 piglets were re-suspended and serially diluted (10^−2^ ~ 10^−5^) in pre-reduced PBS. Dilution (100 µL) was spread evenly on de man, Rogosa, Sharpe (MRS, CM1175, Oxoid, USA) agar and incubated at 37 °C in an anaerobic chamber for 24 h prior to colony selection. Pure cultures were obtained by re-streaking single colonies at least three times before transferring into MRS broth in Hungate tubes. DNA was extracted from pure culture and 16S full-length rRNA was amplified and sequenced as described previously [16]. Sequences were classified using EzBioCloud [17]. Growth curve was determined for isolated strains. Isolated strains were used in the cell and mouse culture experiments (Additional file 3: Fig. S1).

### Antibiotic cocktail (ABX) treatment

After 1 week of acclimation, an antibiotic cocktail (1 g/L streptomycin, 0.5 g/L ampicillin, 1 g/L gentamicin, and 0.5 g/L vancomycin) was fed daily to SPF mice for 2 weeks. To avoid confounding effects from chronic stress induced by oral gavage, antibiotics were administered in drinking water. At the end of the bacterial clearance, q-PCR tests were used to determine total bacteria count in the feces. All antibiotics were purchased from Meilun Bio (Dalian, China).

### Fecal microbiota transplantation (FMT)

Feces of the donor pigs (CF group, CR group) was collected, homogenized, and diluted threefold in sterile PBS containing 15% glycerol (v/v). Samples were centrifuged at 100 × *g* for 5 min to remove large diet particles. A total of 200 μL of supernatant was administered to ABX mice via oral gavage daily for 2 weeks. Where noted, the FMT suspension of CR-Lj group was obtained by resuspending *L. johnsonii* with the CR suspension.

### Histological analysis

For morphological measurements, proximal colon tissues were consecutively sectioned at 5 μm thickness and stained with hematoxylin and eosin (H&E). Scanning images were evaluated using the Image Viewer v3.2 software. The extent of inflammatory infiltration, histopathological changes in crypt structure, ulceration and crypt loss, ulcer, and presence of edema were measured. Histological scores were determined as described previously [18]. To count colonic goblet cells, Carnoy’s fixed colonic tissues were stained in Alcian blue following manufacturer’s instructions (Servicebio, Wuhan, China). Briefly, sections were stained with Alcian blue solution for 10 to 15 min, and then were rinsed with distilled water. Slides were dehydrated with absolute ethyl alcohol and xylene followed by image acquisition on slide scanner VENTANA DP 200 (Roche, USA). Goblet cell counts were calculated as the average of the number of goblet cells found in 10 randomly chosen intact crypts per mouse.

### RNA preparation and analysis

RNA was isolated from cultured cells and colonic tissues using TRIzol Reagent (15,596,026, Invitrogen, Life Technologies, USA) and quantified using a Nanodrop 2000. Reverse transcript reaction was performed using 1 μg RNA with a reverse transcript enzyme (MF166-plus-01, Mei5bio, China). q-PCR was detected on an LightCycler 96 (Roche, USA) using a standard protocol. The primer sequences are listed in Additional file 3: Table S3. *β-actin* was used for data normalization.

### Quantification of bacterial load by q-PCR

The quantification of target bacteria from colonic digesta was determined using q-PCR analysis. The procedure of q-PCR analysis was same as mentioned above. Preparation of template DNA containing the 16S rRNA genes was referred to previous description [19]. Standard plasmids were constructed by cloning the gene of probes into the pUC57 vector and used as template DNA for q-PCR analysis. The copy numbers of each target bacteria were quantified according to the corresponding standard curves. Standard curves were generated by a series of tenfold dilution (10^9^ to 10^1^ copies/μL) of plasmid DNA concentrations and Cq values for each dilution. The gene copy numbers were calculated using the following equation: [plasmid DNA concentration (μg/μL) × 6.0233 × 10^23^ copies/mol] / [DNA size (bp) × 660 × 10^6^]. These primers for total bacteria, total *Lactobacillus*, and *L. johnsonii* are listed in Additional file 3: Table S4. The measured Cq value exceeded 33.8 meant not detected.

### Immunofluorescence

Colonic tissue was fixed in cold 4% paraformaldehyde, and then the samples were dehydrated in ethanol and toluene, embedded in paraffin, and sectioned at 5 μm thickness. Samples were transferred to microscope slides treated with 3-aminopropyl-triethoxysilane-treated for immunofluorescence staining. Briefly, sections were deparaffinized and rehydrated, and antigen retrieval was performed by exposure to microwaves for 15 min in 0.01% sodium citrate buffer (pH 6.0). Sections were blocked with 10% normal donkey serum and incubated overnight at 4 °C with primary antibodies (Abcam, UK) against Ki67 (1:200, ab15580), MUC2 (1:200, ab272692), ZO-1 (1:200, ab221547), or E-cadherin (1:200, ab231303). Next, sections were incubated with Alexa Fluor 488- or 555- conjugated secondary antibodies (1:100, Invitrogen, Life Technologies, USA) for 1 h at 37 °C and Hoechst 33,342 (B2261; Sigma Aldrich, USA) as a nuclear counterstain. Samples were observed under an A1 confocal laser microscope (Nikon, Japan).

### Western blotting

Total protein of the colon was isolated using RIPA lysis buffer (R0010, Solarbio, China) with a protease inhibitor cocktail. Total protein was quantified using a bicinchoninic acid (BCA) protein assay kit (23,227, Thermo Fisher Scientific, USA). Equal amounts of protein (300 ng) were loaded on sodium dodecyl sulfate–polyacrylamide gel electrophoresis and then transferred to a polyvinylidene fluoride membrane (IPVH00010, Millipore, USA). Membranes were blocked with 5% (w/v) skimmed milk and incubated against primary antibodies (β-actin, MUC2, ZO-1, and E-cadherin, 1:1000, Abcam, UK) overnight, followed by incubation against the corresponding DyLight 800-labeled anti-mouse IgG antibody (1:1000, Cell Signaling Technology, USA) and anti-rabbit IgG antibody (1:1000, Cell Signaling Technology, USA). Blots were detected with an Odyssey Clx (4647 Superior Street, LI-COR Biotechnology, NE). Band density was quantified after normalization to β-actin using ImageJ v1.8.0 software.

### DNA extraction, library preparation and shotgun metagenomic sequencing

Total genomic DNA was extracted from day 28 pigs colonic digesta or feces samples using E.Z.N.A Soil DNA kit (D5625, Omega Bio-tec, USA). Concentrations and purity of DNA were determined with TBS-380 and NanoDrop2000 (Thermo Fisher Scientific, USA), respectively. Quality of extracted DNA was checked on 1% agarose gel. DNA was then fragmented to approximately 400 bp using Covaris M220 (Gene Company Limited, China). Adapter ligation, cleanup, and enrichment were performed using NEXTFLEX Rapid DNA-Seq (Bioo Scientific, USA). Shotgun metagenomic sequencing was performed on Illumina NovaSeq 6000 at Majorbio Bio-Pharm Technology Co., Ltd. (Shanghai, China). Sample information of experiments is listed in Additional file 2: Table S2.

### Quality control, host decontamination

Raw sequencing data was filtered to remove low-quality reads and adapter using fastp v0.19.4 with parameters “–cut_by_quality3 -W 4 -M 20 -n 5 -c -l 150 -w 3” [20]. Filtered reads were then mapped to pig genome to remove host contamination using bowtie2 v2.4.1 [21]. The resulting high-quality clean reads were used for downstream analysis.

### Calculation of taxonomic abundance

Metaphlan3 v3.0.7 was used to perform reads-based taxonomy classification and abundance calculation [22]. MetaPhlAn3 relies on approximate 1.1 M unique clade-specific marker genes identified from around 100,000 reference genomes (99,500 bacterial and archaeal and 500 eukaryotic), allowing unambiguous taxonomic assignments, accurate estimation of organismal relative abundance, and species-level resolution for bacteria, archaea, eukaryotes, and viruses.

### DNA extraction, 16S sequence processing and analysis

Microbial DNA was extracted from mice colonic digesta samples using FastDNA SPIN Kit for Soil (MP Biomedicals, USA). Concentrations and quality of DNA were assessed using Nanodrop2000 and 1.5% agarose gel electrophoresis. V3–V4 regions of the bacteria 16S rRNA gene were amplified with primer pairs 338F (5′-ACTCCTACGGGAGGCAGCAG-3′) and 806R (5′-GGACTACHVGGGTWTCTAAT-3′) by an ABI GeneAmp 9700 PCR thermocycler (ABI, USA). PCR products were purified, quantified, pooled, and sequenced on the Illumina MiSeq PE300 platform (Illumina, USA) according to the standard protocols by Majorbio Bio-Pharm Technology Co. Ltd.

16S raw sequencing reads were demultiplexed according to sample-specific barcode (6–8 nucleotides) and imported into the QIIME2 platform v2020.2 [23]. Quality control and denoising were performed simultaneously using DADA2 with default parameters to generate Amplicon sequence variants (ASVs) [24]. Only ASVs with a minimum abundance of two reads and detected in more than two samples were retained. The phylogenetic tree was generated using the SEPP algorithm against the silva 132 database with default parameters [25]. To avoid bias resulting from different sequencing depths, all samples were rarefied to 6244 sequences [26]. All ASVs were classified against the silva 132 database by naïve Bayes classifier constructed by the scikit-learn software [27]. α- and β-diversity were calculated using the vegan package v2.5–6 inside the R software. PCoA was performed using weighted Bray–Curtis and UniFrac distance metrics. PERMANOVA was used to evaluate factors shaping microbiota by using the adonis function of the “vegan” package (999 permutations). Differential taxa were identified by LEfSe (linear discriminant analysis effect size) and further classified against the NCBI 16S rRNA database using blast software [28].

### Quantification of SCFAs

SCFAs (i.e., lactate, acetate, propionate, butyrate) in colonic contents were quantified the procedures of Wang et al. [29]. Briefly, pig samples were thawed on ice and approximately 0.5-g samples were added to 8 mL of deionized water (For mouse samples: 50 mg of samples was added to 1 mL of deionized water). The mixture was thoroughly homogenized by vortexing for 1 min and centrifuged at 13,000 × *g* for 5 min. The supernatant was diluted 50-fold for pig samples or 25-fold for mouse samples and filtered through a 0.22-μm filter (Millipore, USA). The solution was transferred into the vial for quantitative determination using ion chromatography (ICS-3000, Dionex, USA) with a Dionex Ionpac**™** AS11 analytical column (4 × 250 mm). To determine SCFA concentrations of the bacterial media, 1 mL media was collected at each time point and centrifuged at 13,000 × *g* for 5 min. The supernatant was diluted 100-fold and filtered through a 0.22-μm filter for ion chromatography analysis.

### Copper content determination

Copper contents in jejunal, colonic, and fecal digesta were determined using inductively coupled plasma-mass spectroscopy 7500 (ICP-MS, Agilent, USA). Briefly, samples were digested by 98% HNO_3_ and Milli-Q water (V:V = 1:1) at 100 °C for about 1 h. After centrifugation (3000 rpm, 5 min), supernatants were collected for determination of copper content by ICP-MS.

### Statistical analysis

Statistical analysis was performed using the R software v3.6.3 (https://www.r-project.org/) or GraphPad Prism v9.3.1. For the two group comparisons, Student’s two-tailed unpaired *t* test was used. For the others, one-way ANOVA followed by multiple comparisons testing (Turkey’s test). α-diversity was analyzed using Kruskal–Wallis test. PCoA was analyzed employing PERMANOVA.

## Results

### Positive effects of copper at the cost of compromised colonic barrier

**Fig. 1:**
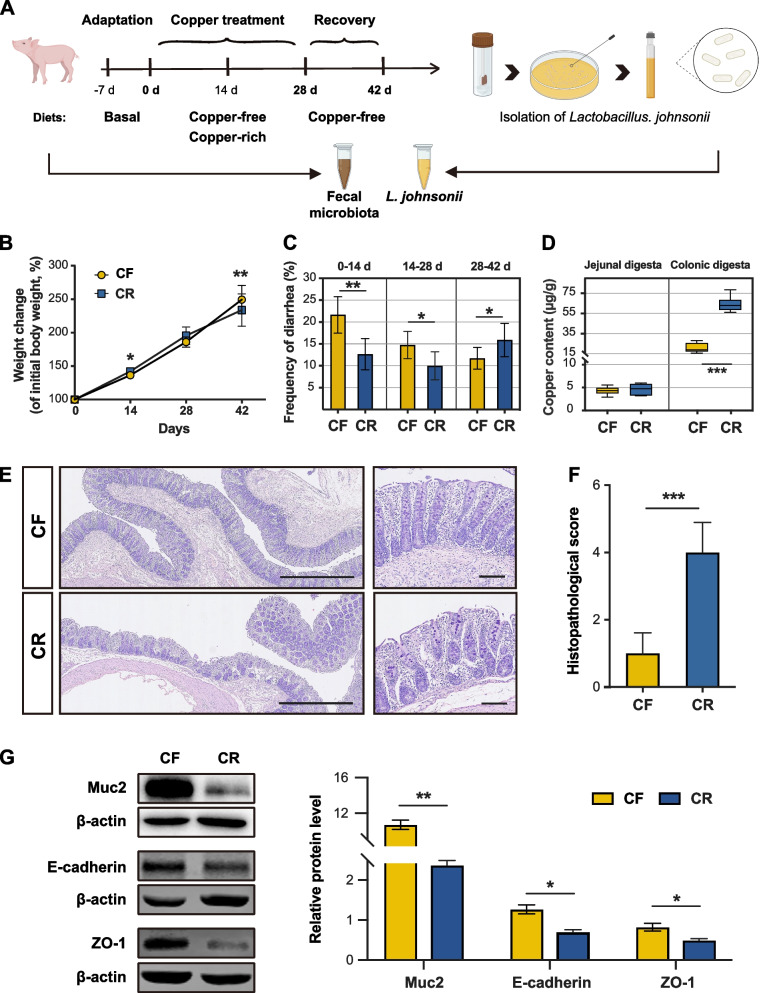
The positive effects of high copper are accompanied by colonic damage. **A** Schematic of the weaned piglet experiment. **B** Weight change in piglets fed different diets. **C** Frequency of diarrhea when fed different diets. *n* = 36 piglets/group. **D** Copper contents within jejunal and colonic digesta. **E** H&E-stained colonic sections of two groups. **F** Histopathological scores of colons. *n* = 6 piglets/group. **G** Western blot of MUC2, E-cadherin, and ZO-1 in the colon. CF copper-free, CR copper-rich. Values are shown as means and error bars represent SEM. Statistical significance was determined using a two-tailed *t* test. *p ≤ 0.05, **p ≤ 0.01, ***p ≤ 0.001

In the present study, we profiled the effects of copper on weaned piglets including growth performance, intestinal barrier function, SCFA concentrations, and gut microbiome community. Piglets in CR group showed a greater weight gain during 0 to 14 days (Fig. 1B) than CF pigs. However, after switching to the copper-free diet at day 28, the CR group greatly reduced their rate of weight gain (Fig. 1B). Similarly, CR group had a lower frequency of diarrhea during 0 to 28 days, but incidence of diarrhea spiked in the CR group after day 28 (Fig. 1C). Copper mainly accumulated in colonic digesta, fourfold as in the jejunal digesta. Besides, in the colonic digesta, the copper content in CR group was threefold as CF group (Fig. 1D). After switching to the copper-free diet, there were no significant differences in fecal copper content in both groups on day 42 (Additional file 4: Fig. S2). Histological examination revealed more extensive bowel edema in the mucus layer, more loosely arranged intestinal glands, greater intestinal villus structure damage, and inflammatory cell infiltration in colon of CR group compared with CF pigs, and histological score also confirmed these differences (Fig. 1E and F). Similarly, protein expression of ZO1, E-cadherin, and MUC2 (Fig. 1G) and mRNA expression of barrier markers (*Claudin1*, *ZO-1*, *E-cadherin*, *Occludin*, and *MUC2*, Additional file 5: Fig. S3) were decreased in CR group. These results revealed that the positive effects of high copper are accompanied by colon barrier damage.

### High copper leads to extinction of *L. johnsonii*

**Fig. 2:**
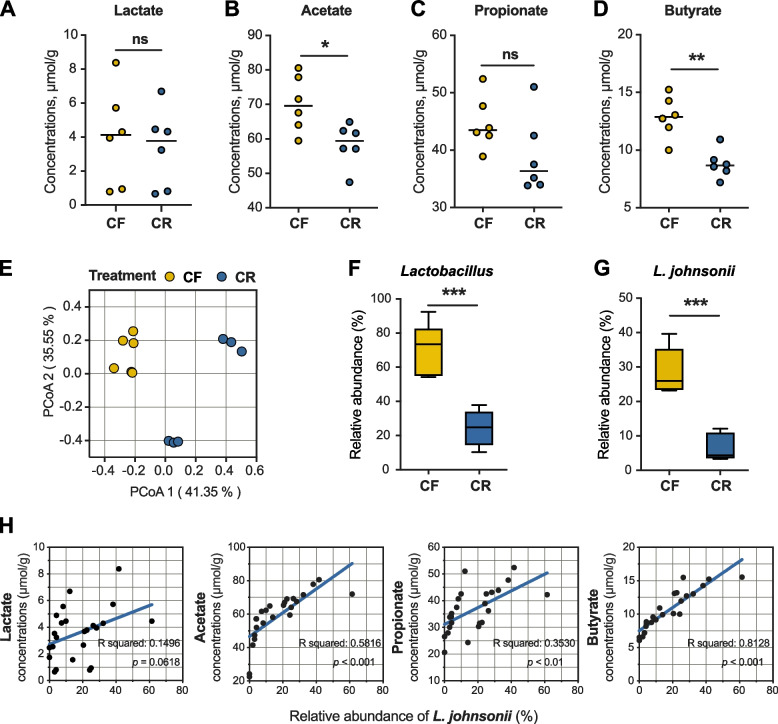
Responses of colonic microbiome and SCFAs to dietary copper supplementation. Concentrations of colonic lactate (**A**), acetate (**B**), propionate (**C**), and butyrate (**D**). **E** PCoA of the colonic microbiome based on weighted UniFrac distance metrics. Significance between community structure was evaluated by PERMANOVA. Relative abundance of *Lactobacillus* (**F**) and *L. johnsonii* (**G**) in the colon. *n* = 6 piglets/group. **H** SCFA (lactate, acetate, propionate, butyrate) concentration and its association with relative abundance of *L. johnsonii* within the colonic digesta and feces of piglets on day 28. CF copper-free, CR copper-rich. Values are shown as mean and error bars represent SEM. Statistical significance was determined using two-tailed *t* test. *p < 0.05, **p < 0.01, ***p < 0.001

No significant differences in lactate and propionate concentrations were detected among CF and CR groups (Fig. 2A and C) on day 28 of the experiment. A significant decrease in acetate and butyrate concentrations was observed in the CR group (Fig. 2B, D). Obvious separation for CF and CR pigs was observed in community structure for both colon and feces on day 28 (PERMANOVA, *P* < 0.05, Fig. 2E and Additional file 6: Fig. S4A). At the phylum level, Firmicutes dominated both the colonic digesta and feces on day 28, accounting for over 88% relative abundance in CF group (Additional file 6: Fig. S4B and C). In contrast, relative abundance of Firmicutes decreased to about 50% in CR group (Additional file 6: Fig. S4B and C). Copper supplementation resulted in the extinction of *Lactobacillus* of colonic digesta and feces on day 28, especially *L. johnsonii* (Fig. 2F, G, and Additional file 6: Fig. S4D and E). Absolute counts of *Lactobacillus* and *L. johnsonii* within colon also showed the same change pattern with their relative abundance, which decreased in CR group compared to CF group (Additional file 6: Fig. S4H). LEfSe analysis at the species level was applied to find bacteria biomarkers discriminating copper-free and copper-rich diets with LDA score threshold set at 4.0 and *L. johnsonii* was identified as the key differential species between CF and CR groups (Additional file 6: Fig. S4F and G). In addition, the abundance of *L. johnsonii* exhibited a positive correlation with concentrations of acetate, propionate, and butyrate (Fig. 2H). Collectively, *L. johnsonii* extinction might play an important role in the process of copper-induced colonic barrier.

### *L. johnsonii* protects enterocyte barrier against copper exposure in vitro

To further explore the effects of *L. johnsonii* on copper-induced colon damage, we isolated 13 strains of *L. johnsonii* from the feces in CF group and performed in vitro screening. We selected the top three strains (*L. johnsonii* YXY11, YXY12, YXY13) based on the results of TEER (Additional file 7: Fig. S5A). To screen for the strains with the maximum protective capacity under copper stress, we measured the mRNA expression of tight junction protein and cell viability. The *Claudin1*, *ZO1*, *E-cadherin*, and *Occludin* gene expressions in CS group were significantly downregulated compared to CON group. However, the negative effects of copper damage were alleviated by incubating with co-incubation with *L. johnsonii* (Additional file 7: Fig. S5D). In line with the mRNA expression, decreased cell viability was also reversed by *L. johnsonii* (Additional file 7: Fig. S5C). Collectively, *L. johnsonii* YXY13 could protect the barrier function well against copper exposure. Therefore, YXY13 was used for all subsequent animal experiments.

### *L. johnsonii* reverses high-copper-induced colonic barrier damage

**Fig. 3:**
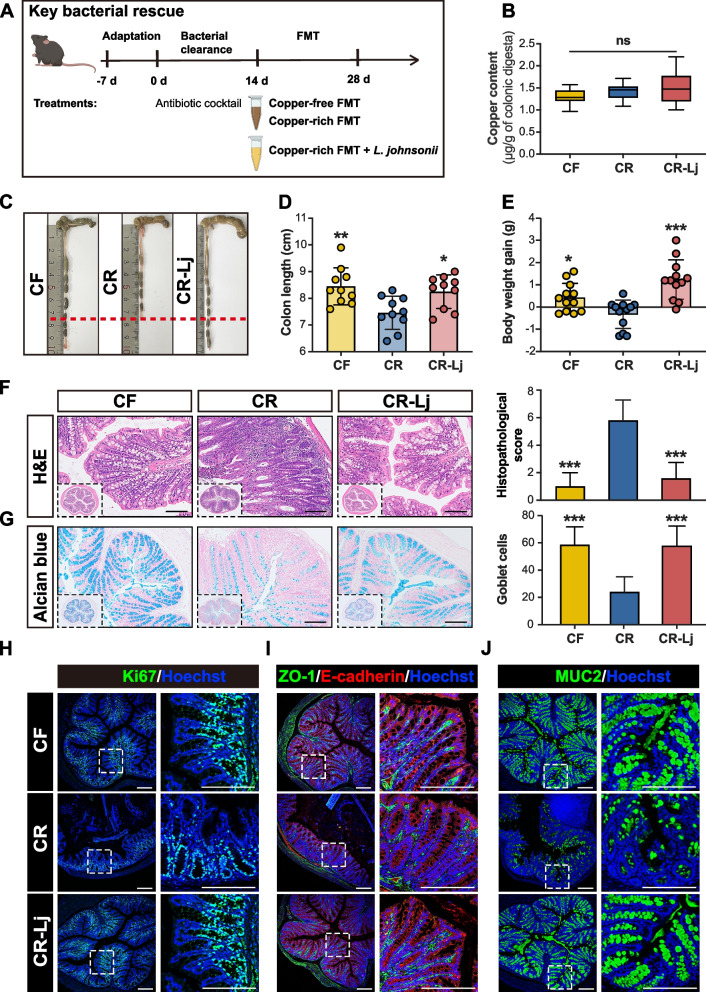
Transplanting fecal microbiota to ABX mice reproduced the gut characteristics of pig donors. **A** Schematic of the experiment for key bacterial rescue. **B** Copper contents within colonic digesta. Macroscopic images of representative colons (**C**) and colon length (**D**). **E** Body weight gain in mice following FMT and *L. johnsonii* administration. **F** Representative images of colon H&E staining, and its histopathological scores of colons. *n* = 8 ABX mice/group. **G** Images of representative Alcian blue-stained colonic sections and its goblet cell numbers per crypt. Representative immunofluorescence images of colonic sections stained with Ki67 (**H**), ZO-1 and E-cadherin (**I**), MUC2 (**J**) antibody, and Hoechst. CF, fecal bacterial suspension of pigs in copper-free group. CR, fecal bacterial suspension of pigs in copper-rich group. CR-Lj, fecal bacterial suspension of pigs in copper-rich group with *L. johnsonii*. Values are shown as mean and error bars represent SEM. One-way ANOVA with Tukey’s test, CR group versus other groups. *p < 0.05, **p < 0.01, ***p < 0.001

To investigate whether *L. johnsonii* extinction contributes meaningfully to copper-induced colon damage, we performed a FMT experiment with mice. The antibiotic cocktail was extremely effective in cleaning bacteria from the gut of mice (Additional file 8: Fig. S6B). The absolute counts of *L. johnsonii* revealed that *L. johnsonii* was successfully colonized in the colon of the CR-Lj group (Additional file 8: Fig. S6C). We successfully reproduced the growth performance and gut characteristics of the pig donors in ABX mice through FMT. Negative body weight gain and colonic damage were observed in CR group (Fig. 3). To isolate the effect of microorganisms, we then excluded the distracting factor of copper content by measured the colonic copper content in mice. No difference in colonic copper content was detected between CF and CR groups (Fig. 3B). The results confirmed that microbiota mediated copper-induced damage of the colon.

Specifically, the CR-Lj group showed greater weight gain compared to CR group (Fig. 3E). Decreased colon length and protein expression of Ki67 were observed in the CR group, but recovered to normal with *L. johnsonii* administration (Fig. 3C, D, and H). Similarly, *L. johnsonii* rescue significantly reversed copper-induced mucosal erosion. Colonic histology scores were also consistent with the histological analysis (Fig. 3F). Goblet cells and MUC2 protein expression in CR group were significantly decreased compared to CF group, *L. johnsonii* administration restored it to normal (Fig. 3G, J and Additional file 8: Fig. S6A). Similarly, the protein expressions of ZO-1 and E-cadherin in CR-Lj group were significantly higher than that in CR group (Fig. 3I and Additional file 8: Fig. S6A). The protective effect of *L. johnsonii* further validates that microbiota mediate copper-induced damage of colonic barrier.

### *L. johnsonii* restores SCFA function in a microbiota-dependent manner

**Fig. 4:**
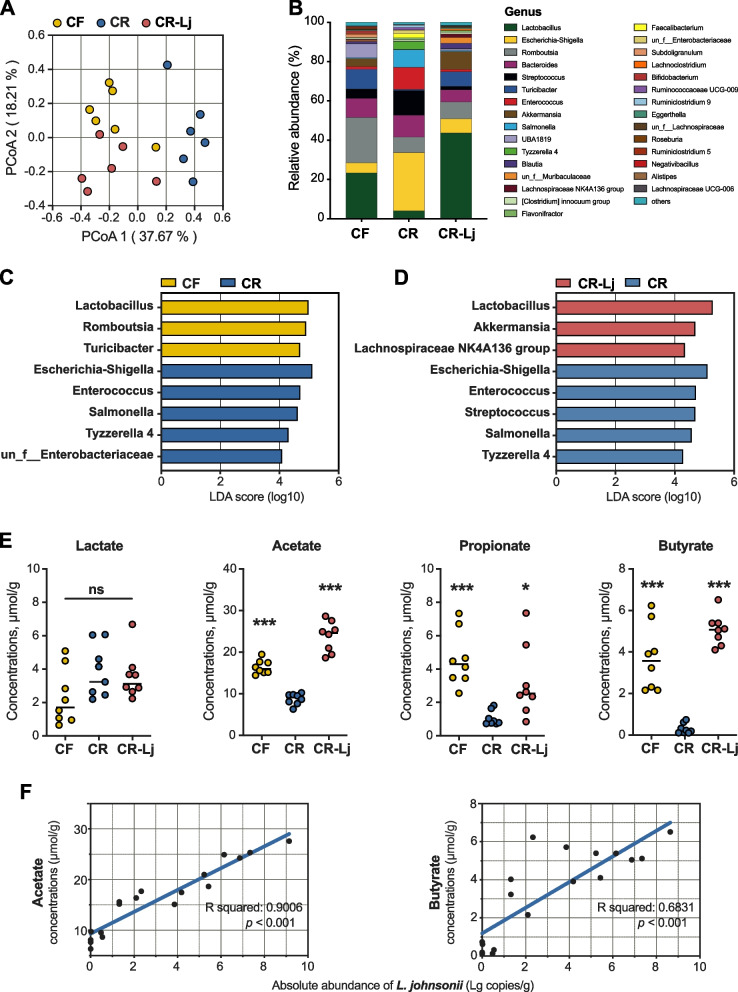
SCFAs and microbial community following *L. johnsonii* administration. **A** PCoA of the colonic microbiome based on weighted the UniFrac distance metrics. Significance between community structure was evaluated by PERMANOVA. **B** The mean relative abundance of top 30 genera in the colon. The most differential taxa at genus level were exhibited by LEfSe analysis; CR group versus CF group (**C**), CR group versus CR-Lj group (**D**). **E** Concentrations of colonic lactate, acetate, propionate, and butyrate. **F** SCFA (acetate, butyrate) concentration and its association with copy number of *L. johnsonii* within the colonic digesta of mice on day 28. CF, fecal bacterial suspension of pigs in copper-free group. CR, fecal bacterial suspension of pigs in copper-rich group. CR-Lj, fecal bacterial suspension of pigs in copper-rich group with *L. johnsonii*. Values are shown as mean and error bars represent SEM. One-way ANOVA with Tukey’s test, CR group versus other groups. *p < 0.05, ***p < 0.001

We further explored the change of colon microbial communities among treatment groups using 16S rRNA amplicon sequencing. No significant difference in Shannon index of colonic microbiota was observed (Additional file 8: Fig. S6D). However, observed species was significantly decreased in the CR group (Additional file 8: Fig. S6E). In addition, the distinct separation between CR-Lj and other two groups were observed by PCoA (PERMANOVA, *P* < 0.05, Fig. 4A). At the phylum level, Firmicutes, Proteobacteria, and Bacteroidetes dominated in the CR group, accounting for 99% relative abundance (Additional file 8: Fig. S6F). For CF and CR-Lj groups, Firmicutes dominated the colon, accounting for over 67% of relative abundance (Additional file 8: Fig. S6F). In line with the changes of observed species, the relative abundance of *Lactobacillus* particularly decreased in the CR group at the genus level (Fig. 4B) similar to what we observed in pig donors. Moreover, we found that multiple pathogenic bacterial genera and markers of colitis were mainly enriched in the CR group including *Escherichia-Shigella*, *Streptococcus*, *Enterococcus*, and *Salmonella*, and the administration of *L. johnsonii* ameliorated it (Additional file 8: Fig. S6G). Additionally, it was surprised to find that *L. johnsonii* rescue restored the enrichment of *Lactobacillus*, and promoted growth of *Akkermansia* and *Lachnospiraceae NK4A136 group* (Additional file 8: Fig. S6H). Differentially abundant colonic bacterial genera were identified by LEfSe analysis (CR vs CF, CR vs CR-Lj). For CR vs CF groups, five bacterial genera (*Escherichia-Shigella*, *Enterococcus*, *Salmonella*, *Tyzzerella* 4, and un_f_*Enterobacteriaceae*) were particularly enriched in the CR group, while three different genera (*Lactobacillus*, *Romboutsia*, and *Turicibacter*) were enriched in the CF group (Fig. 4C). For CR vs CR-Lj groups, *Lactobacillus* (classified as *L. johnsonii*), *Akkermansiaceae*, and *Lachnospiraceae NK4A136 group* were abundant in response to *L. johnsonii* rescue, while other five genera (e.g., *Escherichia-Shigella*, *Streptococcus*, *Enterococcus*) were enriched in the CR group (Fig. 4D).

Given the alteration of gut microbiota, we also measured SCFA concentration. Low concentrations of acetate, propionate, and butyrate were observed in the CR group. Surprisingly, acetate, propionate, and butyrate concentrations were restored completely to the CF group levels in the CR-Lj group (Fig. 4E). The absolute abundance of *L. johnsonii* also exhibited a highly positive correlation with concentrations of acetate and butyrate (Fig. 4F). In addition, we found that *L. johnsonii* produces high concentrations of acetate and lactate via in cell culture (Additional file 9: Fig. S7). These results indicate that SCFAs produced by *L. johnsonii* might be a key factor in repairing the intestinal barrier caused by high-copper feeding.

### Acetate and butyrate are required for the protective effect of *L. johnsonii*

To understand the interactive correlation among *L. johnsonii*, SCFAs, and colonic barrier, we added two treatments based on the *L. johnsonii* rescue experiment: (1) CR-A group: CR-FMT with substrate acetate, and (2) CR-B group: CR-FMT with product-butyrate.

**Fig. 5:**
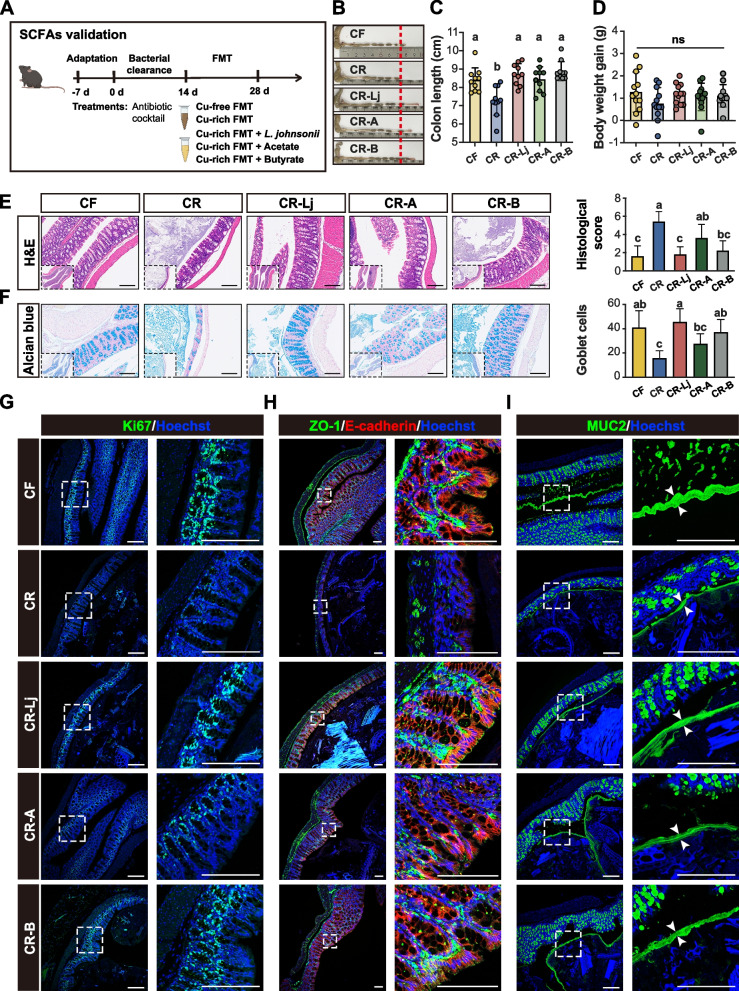
*L. johnsonii* and butyrate administration rather than acetate restore the damaged colon barrier. **A** Schematic of the experiment for SCFAs validation. Macroscopic images of representative colons (**B**) and colon length (**C**). **D** Body weight gain in mice. **E** Representative images of colon H&E staining, and its histopathological scores of colons. *n* = 8 ABX mice/group. **F** Images of representative Alcian blue-stained colonic sections and its goblet cell numbers per crypt. Representative immunofluorescence images of colonic sections stained with Ki67 (**G**), ZO-1 and E-cadherin (**H**), MUC2 (**I**) antibody, and Hoechst. Opposing white arrows with shafts delineate the mucus layer. CF, fecal bacterial suspension of pigs in copper-free group. CR, fecal bacterial suspension of pigs in copper-rich group. CR-Lj, fecal bacterial suspension of pigs in copper-rich group with *L. johnsonii*. CR-A, fecal bacterial suspension of pigs in copper-rich group with acetate. CR-B, fecal bacterial suspension of pigs in copper-rich group with butyrate. Values are shown as mean and error bars represent SEM. One-way ANOVA with Tukey’s test. Statistically significant differences are shown with letters; *p* < 0.05

**Fig. 6:**
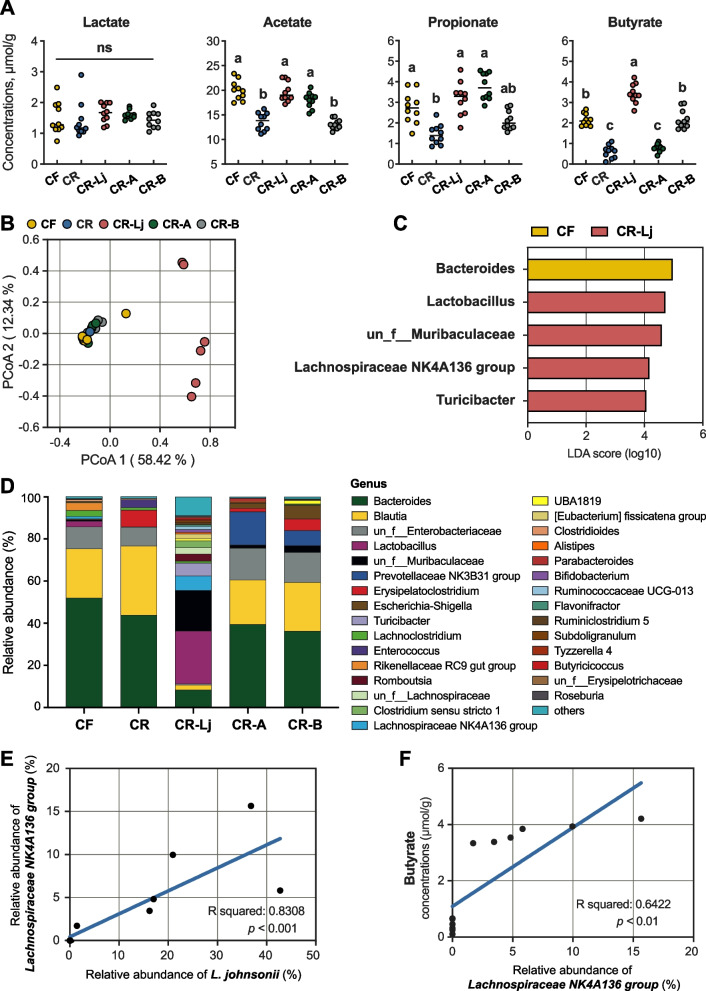
SCFAs and microbial community following acetate and butyrate intervention. **A** Concentrations of colonic lactate, acetate, propionate, and butyrate. **B** PCoA of the colonic microbiome based on weighted the UniFrac distance metrics. Significance between community structure was evaluated by PERMANOVA. **C** The most differential taxa at the genus level were exhibited by LEfSe analysis. **D** The mean relative abundance of top 30 genera in the colonic digesta of mice on day 28. **E** *Lachnospiraceae NK4A136 group* and its association with *L. johnsonii* within the colon. **F** Butyrate concentration and its association with relative abundance of *Lachnospiraceae NK4A136 group* within the colonic digesta of mice on day 28. CF, fecal bacterial suspension of pigs in copper-free group. CR, fecal bacterial suspension of pigs in copper-rich group. CR-Lj, fecal bacterial suspension of pigs in copper-rich group with *L. johnsonii*. CR-A, fecal bacterial suspension of pigs in copper-rich group with acetate. CR-B, fecal bacterial suspension of pigs in copper-rich group with butyrate. Values are shown as mean and error bars represent SEM. One-way ANOVA with Tukey’s test. Statistically significant differences are shown with letters; *p* < 0.05

Consistent with previous experiment for bacterial rescue, the CR-Lj group also showed greater body weight gain, colon length, histological score, goblet cells, protein expressions of colonic barrier, and SCFA concentrations compared to CR group (Fig. 5 and Additional file 10: Fig. S8). Interestingly, there was no significant difference in histological score and goblet cells between CR group and CR-A group, but butyrate administration restored it (Fig. 5E, F). Similarly, Ki67, ZO-1, E-cadherin, and MUC2 protein expression were also significantly upregulated in CR-B group, but partially upregulated in CR-A group (Fig. 5G–I and Additional file 10: Fig. S8A). We further explored the pattern of SCFAs. High acetate concentration but low butyrate concentration was observed in CR-A group (Fig. 6A). In contrary, low acetate and high butyrate concentrations were observed in the CR-B group (Fig. 6A). It is worth mentioning that the butyrate concentration was at a high level in both CR-Lj and CR-B groups. Therefore, we hypothesize that copper-induced gut dysbiosis blocks the butyrate-producing pathway and it leads to disintegration of colonic barrier.

To address the hypothesis, we profiled colonic microbiota composition and specifically investigated changes of bacteria with butyrate-conversion ability. Absolute counts of *L. johnsonii* revealed that *L. johnsonii* was successfully colonized in the CR-Lj colon (Additional file 10: Fig. S8C). α-diversity shown by Shannon index and observed species was mainly impacted by *L. johnsonii* rescue (Additional file 10: Fig. S8D). Furthermore, distinct separation was observed in microbiome structure among the CR-Lj and other groups (Fig. 6B). Combined with Fig. 4, *L. johnsonii* rescue reshaped the colonic microbial environment rather than recovered to that of the CF group. *Lactobacillus* (classified as *L. johnsonii*), *Lachnospiraceae NK4A136 group*, *un_f_Muribaculaceae*, and *Turicibacter* were most enriched mainly in the CR-Lj group (Fig. 6C, D). Some well-known producers of lactate and acetate were also observed in the CR-Lj group, like *Turicibacter* and *Alistipes* (Fig. 6C, D). Given the protective effects of butyrate administration, we examined the higher relative abundance of genera with butyrate-conversion ability and illustrated them in Additional file 10: Fig. S8E. Apparently, *L. johnsonii* rescue promoted microbiota including *Lachnospiraceae NK4A136 group*, *un_f_Lachnospiraceae*, *Flavonifractor*, *Butyricicoccus*, and *Roseburia* colonization in the CR-Lj colon, especially for *Lachnospiraceae NK4A136 group*. However, in the CR-A group, we did not observe the colonization of bacteria with ability to produce butyrate. In addition, we observed a significant linear correlation between relative abundance of *L. johnsonii* and *Lachnospiraceae NK4A136 group* (Fig. 6E). *Lachnospiraceae NK4A136 group* also showed a linear correlation with concentration of butyrate (Fig. 6F). Combined with above observations, *L. johnsonii* rescue resulted in large amounts of butyrate production by providing substrate acetate and mainly promoted *Lachnospiraceae NK4A136 group* to protect colonic barrier.

## Discussion

High copper has been used widely as an inexpensive and highly effective feed additive to promote weight gain and reduce diarrhea in pigs [2]. On the other hand, high copper supplementation also elicits negative effects including environmental pollution and increased antibiotics resistance [4, 5]. Moreover, high copper can induce gut dysbiosis represented by extinction of commensal bacteria and eroding of the mucus barrier [6, 30, 31]. However, copper’s underlying connections are still not unequivocally profiled and validated. In the present study, we first report that high-copper-induced gut dysbiosis is characterized primarily by *L. johnsonii* extinction. We then learned that *L. johnsonii* protects enterocyte barrier function against copper exposure in vitro. Finally, we elucidated that disordered conversion of acetate (produced by *Lactobacillus*) to butyrate is the main mechanism of copper-induced colonic barrier damage.

High-copper supplementation can destroy the structure and function of the intestine [32, 33]. Copper accumulation was also generally recognized as the main factor resulting in high-copper-induced intestinal barrier damage. This copper interacts directly with intestinal epithelial cells by catalyzing Fenton reactions to generate hydroxyl radicals which are damaging to the intestinal epithelium [34]. In addition, recent researchers suggest that copper accumulation induces mitochondrial stress leading to cell death [35]. However, the colon was a hypoxic environment. It was difficult for copper ions to catalyze ROS production. We hypothesized that copper might damage the colonic barrier in different way. Here, our approach excluded the controversial factor—copper accumulation, and reproduced colonic damage of pig donors in ABX mice through FMT. Therefore, we revealed that microbiota might mediate copper-induced damage of the colonic barrier. In addition, previous researchers reported copper-induced changes in specific microbes, mainly *Lactobacillus* [6, 9, 36], which is consistent with the extinction of *Lactobacillus* found in the present study. Here, our work identified *L. johnsonii* as the marker species using shotgun metagenomic sequencing and LEfSe analysis. Others reported that *L. johnsonii* can effectively alleviate decreased expression of tight junction proteins, and thinning of the mucus layer induced by colitis in mice [37, 38]. At present, we isolated *L. johnsonii* from pig feces and supplemented it to ABX mice with copper-rich FMT. Surprisingly, *L. johnsonii* administration reversed colonic damage caused by high-copper feeding, which further established a central role for gut microbiota in copper-induced colon damage. However, it is still unclear how *L. johnsonii* fulfilled its protective effect on copper-induced colonic damage.

Generally, gut dysbiosis is accompanied with changes in microbiome-derived metabolites. Consistent with a previous study [14], decreased SCFAs accompanied extinction of *Lactobacillus. Lactobacillus* can produce bioactive compounds, consisting of short-chain fatty acids, reuterin, and bile acid, which are essential for maintaining host physiology [39, 40]. Moreover, *Lactobacillus* is generally recognized as a SCFA producer, but its pathway of SCFA production is species-dependent [41–43]. Bacteria generally produce acetate through the pyruvate dehydrogenase complex (PDH) and pyruvate-formate lyase (PFL) [43], but the *L. johnsonii* genome encodes pyruvate oxidase (POX) that could provide both CO_2_ and acetate [44]. In our study, acetate production by *L. johnsonii* was confirmed by in vitro assay. Similarly, we observed that production of acetate was restored after *L. johnsonii* rescue, and there was a positive relationship between abundance of *L. johnsonii* and acetate concentrations. During the metabolism of the pyruvate via pyruvate oxidase pathway, the intermediate product, acetate, is produced in large quantities [45]. Acetate is the main substrate for synthesis of butyrate [46]. Our study found that acetate rescue in copper-rich FMT did not increase the concentrations of butyrate, indicating that conversion of acetate to butyrate was blocked. Butyrate plays important roles in the maintenance of intestinal barrier integrity in various animal models [47, 48]. Production of butyrate in the colon following *L. johnsonii* rescue, or butyrate administration to ABX mice with copper-rich FMT, both alleviated colon damage in the present study. Despite acetate’s known role in signaling protective barrier effects in the intestine [49], acetate rescue did not completely correct the intestinal barrier damage in this study. Based on these observations, we presented that butyrate was the main metabolite responsible for protective effects of *L. johnsonii*. Besides, considering mice in CR-A or CR-B groups received SCFA through drinking water, the part of SCFA is expected to be absorbed from the upper gastrointestinal tract to portal vein, whereas previous studies reported that SCFAs in portal vein are generally used as a fuel by many tissues [50, 51], and it may have contributed to the improvement of the colonic barrier. This positive effect may be indirect. At present, there are no studies reporting that SCFAs in portal vein can maintain directly the colonic barrier. Of note, due to the conserved property of butyrate production, only specific bacteria genome encoded this capacity, such as *Lachnospiraceae*, *Roseburia*, and *Butyricicoccus* [12, 52]. Consistently, our work showed that *L. johnsonii* drove a series of genera with butyrate-conversion abilities, mainly *Lachnospiraceae NK4A136 group*. Moreover, LEfSe analysis and correction analysis in both mouse experiments showed that decreased *Lachnospiraceae NK4A136 group* might be a potential mediator to block butyrate production. These observations suggested to us that blocked conversion of acetate to butyrate might be a key mechanism linked to copper-induced colonic damage. Regrettably, there is a technical limitation to elucidate the interactions between specific species. Previous researchers reported butyrate can maintain PPAR-γ signaling to limit luminal bioavailability of oxygen by driving energy metabolism of colonic epithelial cells towards β-oxidation and maintaining anaerobiosis in the colon. Anaerobiosis drives the microbial community towards a dominance of obligate anaerobes which produce SCFAs and prevent a disordered expansion of pathogenic bacteria [53]. Similarly, we observed pathogenic bacteria (mainly facultative anaerobes, e.g., *Escherichia-Shigella*, *Streptococcus*, *Enterococcus* and *Salmonella*) dominated in the copper-rich FMT group. Moreover, *L. johnsonii* rescue increased concentration of SCFAs, drove enrichment of obligate anaerobes which produced SCFAs (e.g., *Lactobacillus*, *Lachnospiraceae NK4A136 group*, *Flavonifractor*, *Butyricicoccus*, *Roseburia, Turicibacter*, and *Alistipes*), and decreased relative abundance of pathogenic facultative anaerobes. We reasonably hypothesized that high copper induced an aerobic environment. However, *L. johnsonii* rescue maintained anaerobiosis in the colon via the pyruvate oxidase pathway of acetate production, which could produce CO_2_ and consume O_2_. The anaerobic environment drove enrichment of obligate anaerobes (like *Lachnospiraceae NK4A136 group*), production of a large amount of SCFAs. In turn, SCFAs sustain epithelial PPAR-γ-signaling, which cooperatively drives energy metabolism of colonocytes towards β-oxidation of microbiota-derived butyrate to preserve epithelial hypoxia, thereby closing a virtuous cycle maintaining colon health [52].

## Conclusions

**Fig. 7:**
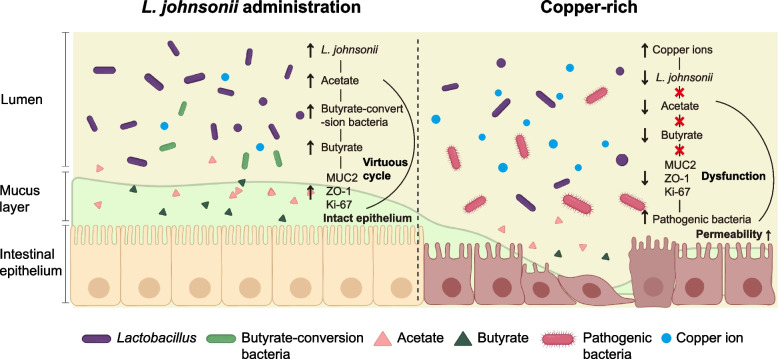
Schematic of the findings. *L. johnsonii* drove the normalization of the butyric acid transformation pathway, leading to homeostasis (*L. johnsonii* administration). In the dietary copper-rich piglet model (Copper-rich), high-copper-induced extinction of *L. johnsonii* and abnormal metabolism of acetate and butyrate, leading to epithelial barrier dysfunction. The schematic diagram was created with BioRender.com

This is the first study to identify a microbiota-mediated mechanism of high-copper-induced colon damage in piglets. Specifically, copper-induced colonic damage is characterized by extinction of *L. johnsonii* accompanied with disordered production of acetate and blocked conversion of acetate to butyrate. In addition, *L. johnsonii* rescue reversed detrimental effects coming from a high-copper diet by providing acetate as substrate for butyrate production. In accordance with this observation, abundance of bacteria with ability to produce butyrate is enriched after *L. johnsonii* administration, mainly *Lachnospiraceae NK4A136 group*. Finally, the protective and SCFA production capacity of *L. johnsonii* were confirmed by in vitro assay. Our proposed mechanisms are summarized in Fig. 7. These findings provide novel insights into the mechanisms of copper-induced colon damage and will facilitate development of therapeutic strategies to mitigate high-copper hazards and other intestinal damage in production practices.

### Supplementary Information

**Additional file 1: Table S1.** Formulation and nutrient composition of experimental diets (%, as-fed basis).

**Additional file 2: Table S2.** Sample information of experiments.

**Additional file 3: Figure S1.** Growth curve (mean OD_600nm_) of *L. johnsonii* YXY13 in MRS medium. **Table S3.** Primer sequences used in RT-qPCR for intestinal barriers. **Table S4.** Primer sequences used for bacterial quantification.

**Additional file 4: Figure S2.** Changes in copper contents of feces in pigs, related to Fig. 1. CF copper-free, CR copper-rich.

**Additional file 5: Figure S3.** The mRNA levels of colonic barrier-related genes, related to Fig. 1. CF copper-free, CR copper-rich. *p ≤ 0.05, **p ≤ 0.01, ***p ≤ 0.001.

**Additional file 6: Figure S4.** Responses of colonic and fecal microbiome to dietary high-copper in pigs, related to Fig. 2. **A** PCoA of the fecal microbiome. The mean relative abundance of phylum in the colon (**B**) and feces (**C**). The mean relative abundance of species in the colon (**D**) and feces (**E**). The most differential taxa at species level were exhibited by LEfSe analysis in the colon (**F**) and feces (**G**). **H** Copy number of total bacteria, *Lactobacillus* and *L. johnsonii* in the colon. CF copper free, CR copper rich. *p < 0.05, **p < 0.01, ***p < 0.001.

**Additional file 7: Figure S5.** *L. johnsonii* could protect enterocyte barrier against copper exposure in vitro. **A** Transepithelial electric resistance (TEER) in epithelial monolayers. **B** Schematic of the experiment for in vitro validation. **C** Cell counting kit 8 (CCK-8) cell viability assay. **D** The mRNA levels of intestinal barrier-related genes. CON control, CS copper sulfate, YXY copper sulfate with *L. johnsonii* YXY. Values are shown as mean and error bars represent SEM. One-way ANOVA with Tukey’s test, CS group versus other groups. *p < 0.05, **p < 0.01, ***p < 0.001.

**Additional file 8: Figure S6.** Microbial community following *L. johnsonii* rescue, related to Figs. 3 and 4. **A** Western blot of ZO-1, Muc2, and E-cadherin in the colon. **B** Copy number of fecal total bacteria in mouse on d 14. *n* = 8 ABX mice/group. **C** Copy number of *L. johnsonii* in the colon on d 28 (limit of detection is 0.064 copies). *n* = 8 ABX mice/group. Alpha diversity as measured by Shannon diversity index (**D**) and Observed species (**E**) in the colonic microbiome. **F** The mean relative abundance of phylum in the colon. The relative abundance of genera enriched in CR group (**G**) and in CR-Lj group (**H**). CF, Fecal bacterial suspension of pigs in copper-free group. CR, Fecal bacterial suspension of pigs in copper-rich group. CR-Lj, Fecal bacterial suspension of pigs in copper-rich group with *L. johnsonii*. Statistical analyses were performed using Kruskal–Wallis test with *P* value adjustment using FDR correction. CR group versus other groups. ***p < 0.001.

**Additional file 9: Figure S7.** Lactate and Acetate concentrations of *L. johnsonii* YXY13 in MRS medium.

**Additional file 10: Figure S8.** Microbial community following acetate, and butyrate intervention, related to Figs. 5 and 6. **A** Western blot of ZO-1, Muc2, and E-cadherin in the colon. **B** Copy number of fecal total bacteria in mouse on d 14. *n* = 8 ABX mice/group. **C** Copy number of *L. johnsonii* in the colon on d 28. *n* = 8 ABX mice/group. **D** Alpha diversity as measured by Observed species and Shannon diversity index in the colonic microbiome. **E** The relative abundance of genera with butyrate-conversion ability. CF, Fecal bacterial suspension of pigs in copper-free group. CR, Fecal bacterial suspension of pigs in copper-rich group. CR-Lj, Fecal bacterial suspension of pigs in copper-rich group with *L. johnsonii*. CR-A, Fecal bacterial suspension of pigs in copper-rich group with acetate. CR-B, Fecal bacterial suspension of pigs in copper-rich group with butyrate. Statistical analyses were performed using Kruskal–Wallis test with *P* value adjustment using FDR correction. One-way ANOVA with Tukey’s test, CR group versus other groups. ***p < 0.001.

## Data Availability

The datasets supporting the conclusions of this article are available in the NCBI Sequence Read Archive (SRA) repository under accession number PRJNA906278 (available on November 29, 2022).

## Abbreviations

SCFA: Short-chain fatty acid
ABX: Antibiotic cocktail
SPF: Specific pathogen-free
FMT: Fecal microbiota transplantation
ICP-MS: Inductively coupled plasma-mass spectroscopy
TEER: Transepithelial electrical resistance
ASV: Amplicon sequence variant
PCoA: Principal coordinates analysis
LEfSe: Linear discriminant analysis effect size
LDA: Linear discriminant analysis
PDH: Pyruvate dehydrogenase complex
PFL: Pyruvate-formate lyase
POX: Pyruvate oxidase
