# Supplementary material for: Blocked conversion of Lactobacillus johnsonii derived acetate to butyrate mediates copper-induced epithelial barrier damage in a pig model
Source: Microbiome. 2023 Sep 30;11:218. doi: 10.1186/s40168-023-01655-2 (PMC10542248; doi:10.1186/s40168-023-01655-2)
Supplement: Supplementary file 2 — Additional file 1: Table S1. Formulation and nutrient composition of experimental diets (%, as-fed basis). [file 40168_2023_1655_MOESM1_ESM.docx]

Table S1. Formulation and nutrient composition of experimental diets (%, as-fed basis)

| Ingredients | % |
| --- | --- |
| Corn | 60.79 |
| Soybean meal | 18.00 |
| Soy protein concentrate | 2.00 |
| Soybean oil | 2.07 |
| Extruded soybean | 8.00 |
| Fish meal, 64.6% | 2.00 |
| Protein whey powder, 3.8% | 4.00 |
| L-Lysine | 0.40 |
| DL-Methionine | 0.09 |
| L-Tryptophan | 0.02 |
| L-Threonine | 0.13 |
| Limestone | 0.90 |
| Dicalcium Phosphate | 0.80 |
| NaCl | 0.30 |
| Premix^a^ | 0.50 |
| Total | 100.00 |
| Nutrient composition (%) |  |
| DE (kcal/kg) | 3499 |
| CP | 18.98 |
| Dig Lys | 1.23 |
| Dig Met | 0.36 |
| Dig Trp | 0.20 |
| Dig Thr | 0.74 |
| Ca | 0.70 |
| P | 0.54 |

^a^ Premix provided the following per kilogram of feed: vitamin A, 12,000 IU; vitamin D, 2,500 IU; vitamin E, 30 IU; vitamin K, 3.0 mg; vitamin B12, 12 μg; D-pantothenic acid, 10 mg; nicotinic acid, 30 mg; choline chloride, 400 mg; Mn, 40 mg; Zn, 100 mg; Fe, 90 mg; I, 0.35 mg; Se, 0.3 mg; The added copper contents were calculated based on copper element, CF group: added 0 mg/kg, measured copper content 5.13 mg/kg; CR group: added 120 mg (Cu) /kg CuSO_4_, measured copper content 124.78 mg/kg.
