# Supplementary material for: Blocked conversion of Lactobacillus johnsonii derived acetate to butyrate mediates copper-induced epithelial barrier damage in a pig model
Source: Microbiome. 2023 Sep 30;11:218. doi: 10.1186/s40168-023-01655-2 (PMC10542248; doi:10.1186/s40168-023-01655-2)
Supplement: Supplementary file 3 — Additional file 2: Table S2. Sample information of experiments. [file 40168_2023_1655_MOESM2_ESM.docx]

Table S2. Sample information of experiments.

| **Experiment 1: experiment for weaned piglet** | | | | |
| --- | --- | --- | --- | --- |
| SampleName | Treatment | Segment | Sequencing | Animal |
| pig_colon_CF1 | CF | Colon | Metagenomics | pig |
| pig_colon_CF2 | CF | Colon | Metagenomics | pig |
| pig_colon_CF3 | CF | Colon | Metagenomics | pig |
| pig_colon_CF4 | CF | Colon | Metagenomics | pig |
| pig_colon_CF5 | CF | Colon | Metagenomics | pig |
| pig_colon_CF6 | CF | Colon | Metagenomics | pig |
| pig_colon_CR1 | CR | Colon | Metagenomics | pig |
| pig_colon_CR2 | CR | Colon | Metagenomics | pig |
| pig_colon_CR3 | CR | Colon | Metagenomics | pig |
| pig_colon_CR4 | CR | Colon | Metagenomics | pig |
| pig_colon_CR5 | CR | Colon | Metagenomics | pig |
| pig_colon_CR6 | CR | Colon | Metagenomics | pig |
| pig_feces_CF1 | CF | Feces | Metagenomics | pig |
| pig_feces_CF2 | CF | Feces | Metagenomics | pig |
| pig_feces_CF3 | CF | Feces | Metagenomics | pig |
| pig_feces_CF4 | CF | Feces | Metagenomics | pig |
| pig_feces_CF5 | CF | Feces | Metagenomics | pig |
| pig_feces_CF6 | CF | Feces | Metagenomics | pig |
| pig_feces_CR1 | CR | Feces | Metagenomics | pig |
| pig_feces_CR2 | CR | Feces | Metagenomics | pig |
| pig_feces_CR3 | CR | Feces | Metagenomics | pig |
| pig_feces_CR4 | CR | Feces | Metagenomics | pig |
| pig_feces_CR5 | CR | Feces | Metagenomics | pig |
| pig_feces_CR6 | CR | Feces | Metagenomics | pig |
| **Experiment 2: experiment for bacterial rescue** | | | | |
| SampleName | Treatment | Segment | Sequencing | Animal |
| fmt_CF1 | CF | Colon | 16S rRNA | mouse |
| fmt_CF2 | CF | Colon | 16S rRNA | mouse |
| fmt_CF3 | CF | Colon | 16S rRNA | mouse |
| fmt_CF4 | CF | Colon | 16S rRNA | mouse |
| fmt_CF5 | CF | Colon | 16S rRNA | mouse |
| fmt_CF6 | CF | Colon | 16S rRNA | mouse |
| fmt_CR1 | CR | Colon | 16S rRNA | mouse |
| fmt_CR2 | CR | Colon | 16S rRNA | mouse |
| fmt_CR3 | CR | Colon | 16S rRNA | mouse |
| fmt_CR4 | CR | Colon | 16S rRNA | mouse |
| fmt_CR5 | CR | Colon | 16S rRNA | mouse |
| fmt_CR6 | CR | Colon | 16S rRNA | mouse |
| fmt_CRM1 | CR-Lj | Colon | 16S rRNA | mouse |
| fmt_CRM2 | CR-Lj | Colon | 16S rRNA | mouse |
| fmt_CRM3 | CR-Lj | Colon | 16S rRNA | mouse |
| fmt_CRM4 | CR-Lj | Colon | 16S rRNA | mouse |
| fmt_CRM5 | CR-Lj | Colon | 16S rRNA | mouse |
| fmt_CRM6 | CR-Lj | Colon | 16S rRNA | mouse |
| **Experiment 3: experiment for SCFAs validation** | | | | |
| SampleName | Treatment | Segment | Sequencing | Animal |
| scfa_CF1 | CF | Colon | 16S rRNA | mouse |
| scfa_CF2 | CF | Colon | 16S rRNA | mouse |
| scfa_CF3 | CF | Colon | 16S rRNA | mouse |
| scfa_CF4 | CF | Colon | 16S rRNA | mouse |
| scfa_CF5 | CF | Colon | 16S rRNA | mouse |
| scfa_CF6 | CF | Colon | 16S rRNA | mouse |
| scfa_CR1 | CR | Colon | 16S rRNA | mouse |
| scfa_CR2 | CR | Colon | 16S rRNA | mouse |
| scfa_CR3 | CR | Colon | 16S rRNA | mouse |
| scfa_CR4 | CR | Colon | 16S rRNA | mouse |
| scfa_CR5 | CR | Colon | 16S rRNA | mouse |
| scfa_CR6 | CR | Colon | 16S rRNA | mouse |
| scfa_CRM1 | CR-Lj | Colon | 16S rRNA | mouse |
| scfa_CRM2 | CR-Lj | Colon | 16S rRNA | mouse |
| scfa_CRM3 | CR-Lj | Colon | 16S rRNA | mouse |
| scfa_CRM4 | CR-Lj | Colon | 16S rRNA | mouse |
| scfa_CRM5 | CR-Lj | Colon | 16S rRNA | mouse |
| scfa_CRM6 | CR-Lj | Colon | 16S rRNA | mouse |
| scfa_CRA1 | CR-A | Colon | 16S rRNA | mouse |
| scfa_CRA2 | CR-A | Colon | 16S rRNA | mouse |
| scfa_CRA3 | CR-A | Colon | 16S rRNA | mouse |
| scfa_CRA4 | CR-A | Colon | 16S rRNA | mouse |
| scfa_CRA5 | CR-A | Colon | 16S rRNA | mouse |
| scfa_CRA6 | CR-A | Colon | 16S rRNA | mouse |
| scfa_CRB1 | CR-B | Colon | 16S rRNA | mouse |
| scfa_CRB2 | CR-B | Colon | 16S rRNA | mouse |
| scfa_CRB3 | CR-B | Colon | 16S rRNA | mouse |
| scfa_CRB4 | CR-B | Colon | 16S rRNA | mouse |
| scfa_CRB5 | CR-B | Colon | 16S rRNA | mouse |
| scfa_CRB6 | CR-B | Colon | 16S rRNA | mouse |
