# Supplementary material for: Blocked conversion of Lactobacillus johnsonii derived acetate to butyrate mediates copper-induced epithelial barrier damage in a pig model
Source: Microbiome. 2023 Sep 30;11:218. doi: 10.1186/s40168-023-01655-2 (PMC10542248; doi:10.1186/s40168-023-01655-2)
Supplement: Supplementary file 4 — Additional file 3: Figure S1. Growth curve (mean OD600nm) of L. johnsonii YXY13 in MRS medium. Table S3. Primer sequences used in RT-qPCR for intestinal barriers. Table S4. Primer sequences used for bacterial quantification. [file 40168_2023_1655_MOESM3_ESM.docx]

Figure S1. Growth curve (mean OD_600nm_) of *L. johnsonii* YXY13 in MRS medium.

Table S3. Primer sequences used in RT-qPCR for intestinal barriers

| Genes | Forward (5′→3′) | Reverse (5′→3′) |
| --- | --- | --- |
| **Pigs** |  |  |
| *β-actin* | CGGGAAATCGTGCGTGACAT | CAGGAAGCAAGGCTGGAAGA |
| *Claudin-1* | AAGGACAAAACCGTGTGGGA | CTCTCCCCACATTCGAGATGATT |
| *ZO-1* | GCCATCCACTCCTGCCTAT | CGGGACCTGCTCATAACTTC |
| *E-cadherin* | CAAACGGCCATTTCAGCTTCA | GTCACCTTGGTGGACAGCTT |
| *Occludin* | CAGCAGCAGTGGTAACTTGG | CAGCAGCAGTGGTAACTTGG |
| *MUC-2* | CTGTGTGGGGCCTGACAA | AGTGCTTGCAGTCGAACTCA |
| **HT-29** |  |  |
| *β-actin* | CACCATTGGCAATGAGCGGTTC | AGGTCTTTGCGGATGTCCACGT |
| *Claudin-1* | GTCTTTGACTCCTTGCTGAATCTG | CACCTCATCGTCTTCCAAGCAC |
| *ZO-1* | GTCCAGAATCTCGGAAAAGTGCC | CTTTCAGCGCACCATACCAACC |
| *E-cadherin* | GCCTCCTGAAAAGAGAGTGGAAG | TGGCAGTGTCTCTCCAAATCCG |
| *Occludin* | ATGGCAAAGTGAATGACAAGCGG  0.59 | CTGTAACGAGGCTGCCTGAAGT  0.59 |

Table S4. Primer sequences used for bacterial quantification

| Bacteria | The sequence for primers (5′-3′) | Product size (bp) | Tm (℃) | Reference |
| --- | --- | --- | --- | --- |
| Total bacteria | F: CGGTGAATACGTTCYCGG | 123 | 60 | Suzuki et al., 2000 |
|  | R: GGWTACCTTGTTACGACT |  |  |  |
| *Lactobacillus* | F: CACCGCTACACATGGAG | 341 | 60 | Su et al., 2008 |
|  | R: AGCAGTAGGGAATCTTCCA |  |  |  |
| *L. johnsonii* | F: CCGCGGCTTAGATTCTGGTA | 115 | 60 | Li et al., 2019 |
|  | R: AGCTAAACGAGCATCTGGGA |  |  |  |

**References**

Su Y, Yao W, Perez-Gutierrez O.N, Smidt H, Zhu W.Y. 16S ribosomal RNA-based methods to monitor changes in the hindgut bacterial community of piglets after oral administration of *Lactobacillus sobrius* S1. Anaerobe. 2007;(14):78-86.

Suzuki M.T, Taylor L.T, Delong E.F. Quantitative analysis of small-subunit rRNA genes in mixed microbial populations via 5'-nuclease assays. Appl Environ Microbiol. 2000;66:4605-4614.

Li N, Huang S, Jiang L, Dai Z, Li T, Han D, Wang J. Characterization of the Early Life Microbiota Development and Predominant *Lactobacillus* Species at Distinct Gut Segments of Low- and Normal-Birth-Weight Piglets. Front Microbiol. 2019;16(10):797.
