# Supplementary figures and images for: Blocked conversion of Lactobacillus johnsonii derived acetate to butyrate mediates copper-induced epithelial barrier damage in a pig model
Source: Microbiome. 2023 Sep 30;11:218. doi: 10.1186/s40168-023-01655-2 (PMC10542248; doi:10.1186/s40168-023-01655-2)

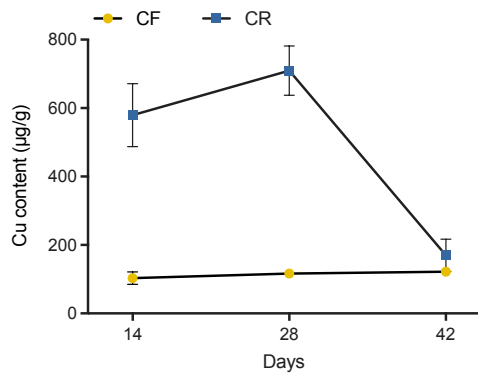

Supplement: Supplementary file 5 — Additional file 4: Figure S2. Changes in copper contents of feces in pigs, related to Fig. 1. CF copper-free, CR copper-rich. [file 40168_2023_1655_MOESM4_ESM.pdf]

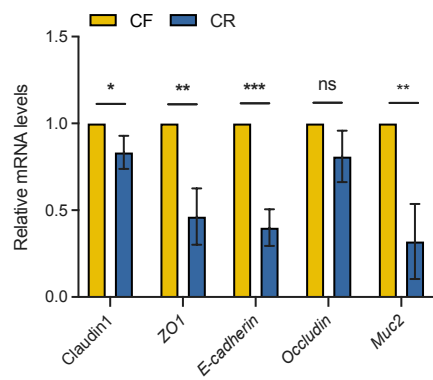

Supplement: Supplementary file 6 — Additional file 5: Figure S3. The mRNA levels of colonic barrier-related genes, related to Fig. 1. CF copper-free, CR copper-rich. *p ≤ 0.05, **p ≤ 0.01, ***p ≤ 0.001. [file 40168_2023_1655_MOESM5_ESM.pdf]

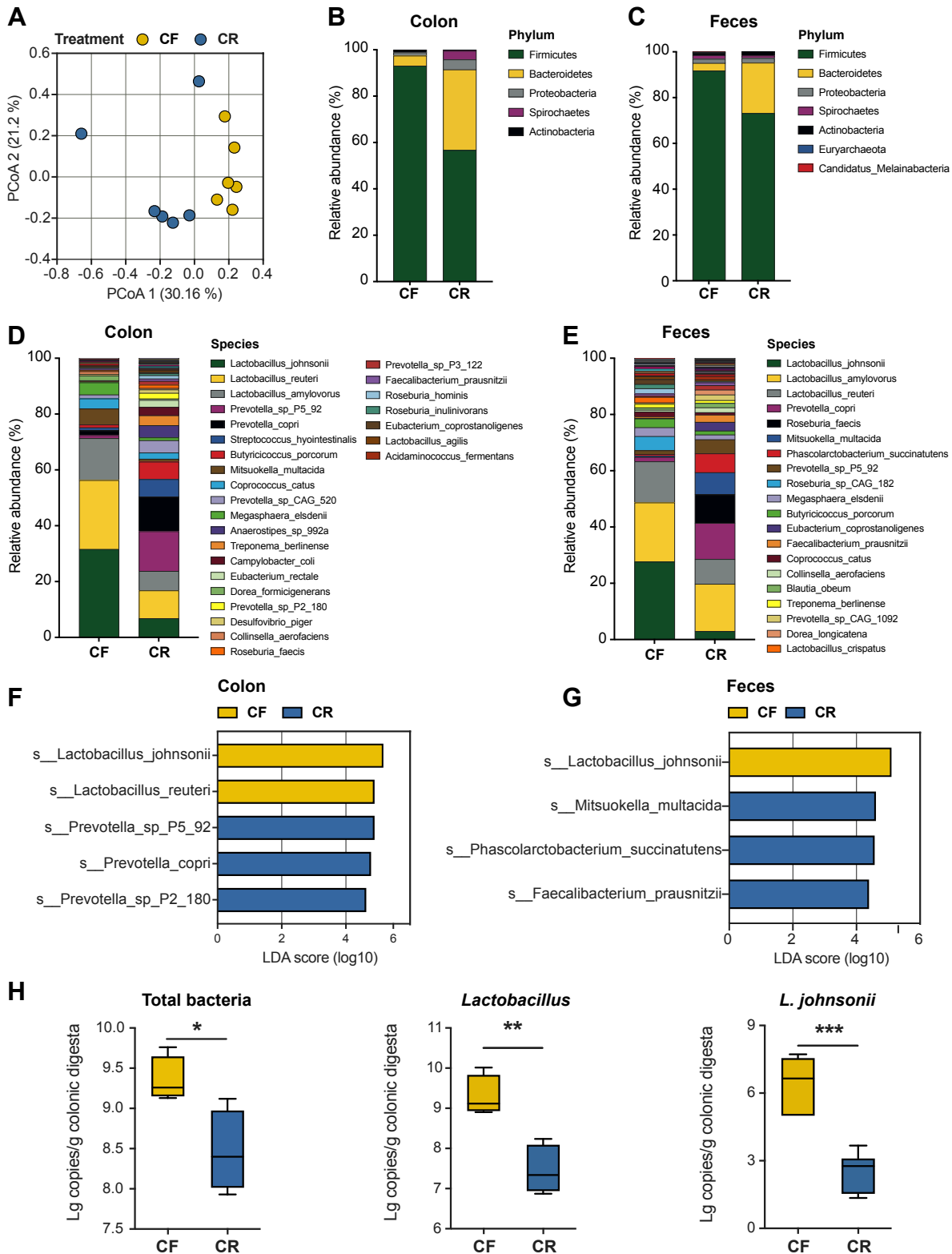

Supplement: Supplementary file 7 — Additional file 6: Figure S4. Responses of colonic and fecal microbiome to dietary high-copper in pigs, related to Fig. 2. A PCoA of the fecal microbiome. The mean relative abundance of phylum in the colon (B) and feces (C). The mean relative abundance of species in the colon (D) and feces (E). The most differential taxa at species level were exhibited by LEfSe analysis in the colon (F) and feces (G). H Copy number of total bacteria, Lactobacillus and L. johnsonii in the colon. CF copper free, CR copper rich. *p < 0.05, **p < 0.01, ***p < 0.001. [file 40168_2023_1655_MOESM6_ESM.pdf]

**A**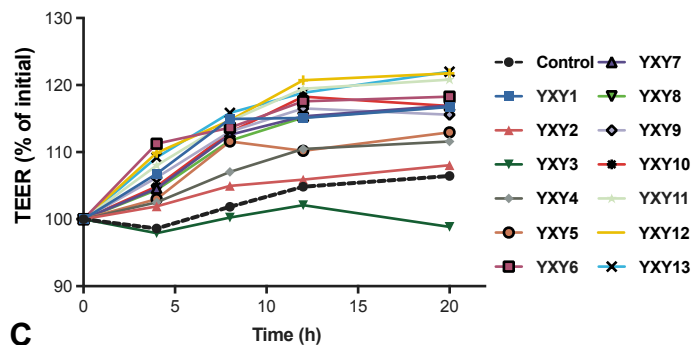**C**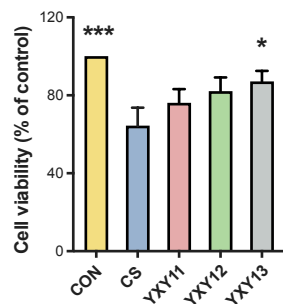**B**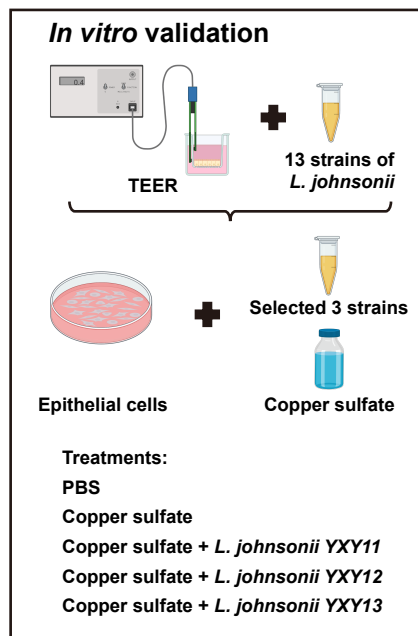**D**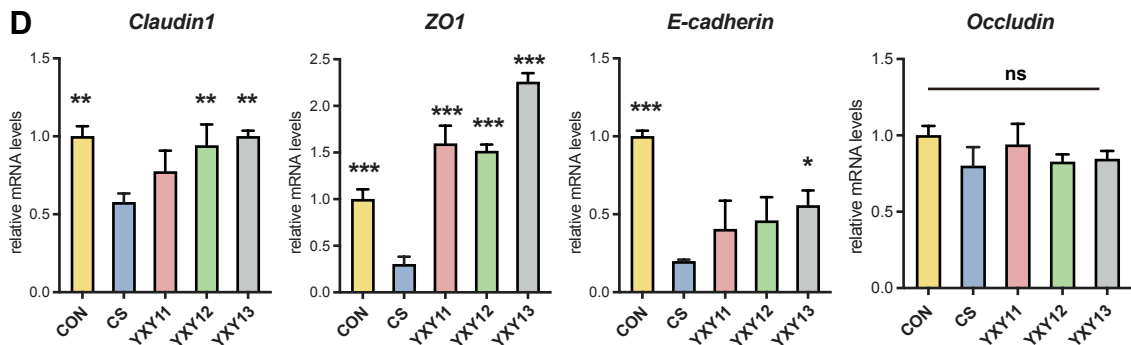

Supplement: Supplementary file 8 — Additional file 7: Figure S5. L. johnsonii could protect enterocyte barrier against copper exposure in vitro. A Transepithelial electric resistance (TEER) in epithelial monolayers. B Schematic of the experiment for in vitro validation. C Cell counting kit 8 (CCK-8) cell viability assay. D The mRNA levels of intestinal barrier-related genes. CON control, CS copper sulfate, YXY copper sulfate with L. johnsonii YXY. Values are shown as mean and error bars represent SEM. One-way ANOVA with Tukey’s test, CS group versus other groups. *p < 0.05, **p < 0.01, ***p < 0.001. [file 40168_2023_1655_MOESM7_ESM.pdf]

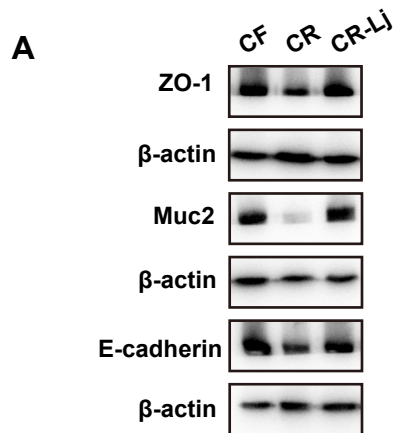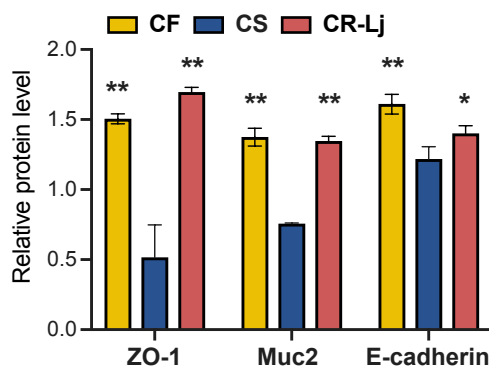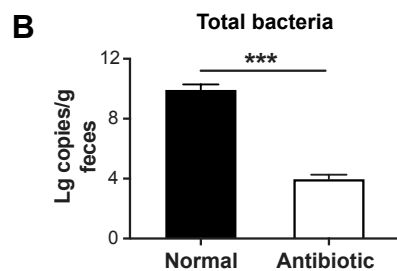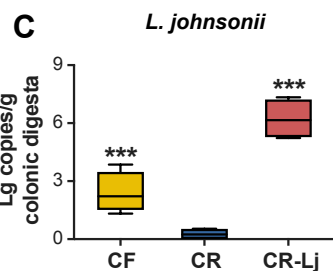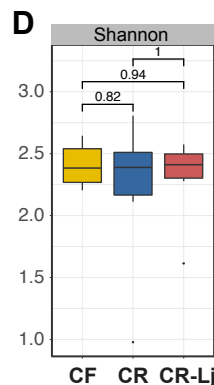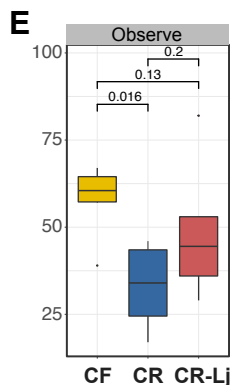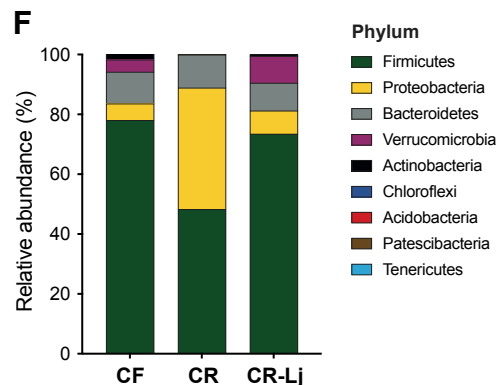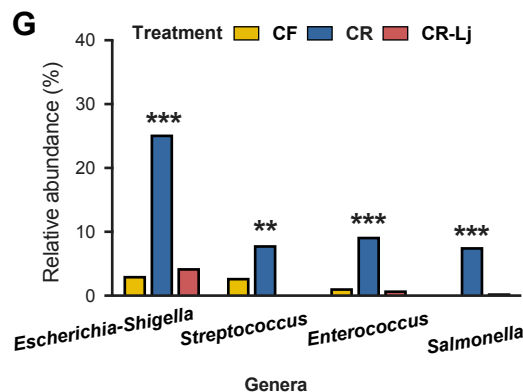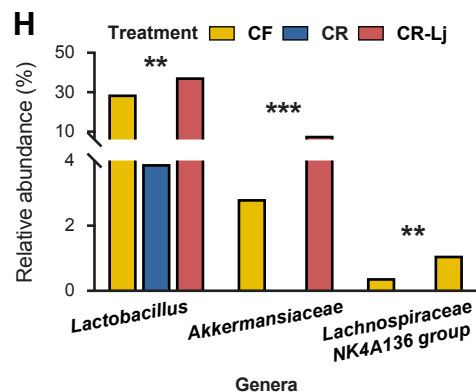

Supplement: Supplementary file 9 — Additional file 8: Figure S6. Microbial community following L. johnsonii rescue, related to Figs. 3 and 4. A Western blot of ZO-1, Muc2, and E-cadherin in the colon. B Copy number of fecal total bacteria in mouse on d 14. n = 8 ABX mice/group. C Copy number of L. johnsonii in the colon on d 28 (limit of detection is 0.064 copies). n = 8 ABX mice/group. Alpha diversity as measured by Shannon diversity index (D) and Observed species (E) in the colonic microbiome. F The mean relative abundance of phylum in the colon. The relative abundance of genera enriched in CR group (G) and in CR-Lj group (H). CF, Fecal bacterial suspension of pigs in copper-free group. CR, Fecal bacterial suspension of pigs in copper-rich group. CR-Lj, Fecal bacterial suspension of pigs in copper-rich group with L. johnsonii. Statistical analyses were performed using Kruskal–Wallis test with P value adjustment using FDR correction. CR group versus other groups. ***p < 0.001. [file 40168_2023_1655_MOESM8_ESM.pdf]

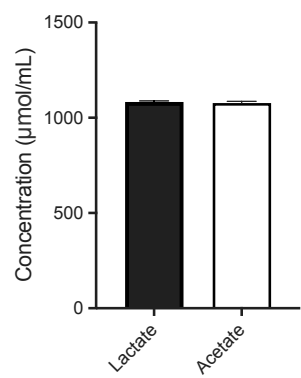

Supplement: Supplementary file 10 — Additional file 9: Figure S7. Lactate and Acetate concentrations of L. johnsonii YXY13 in MRS medium. [file 40168_2023_1655_MOESM9_ESM.pdf]

**A**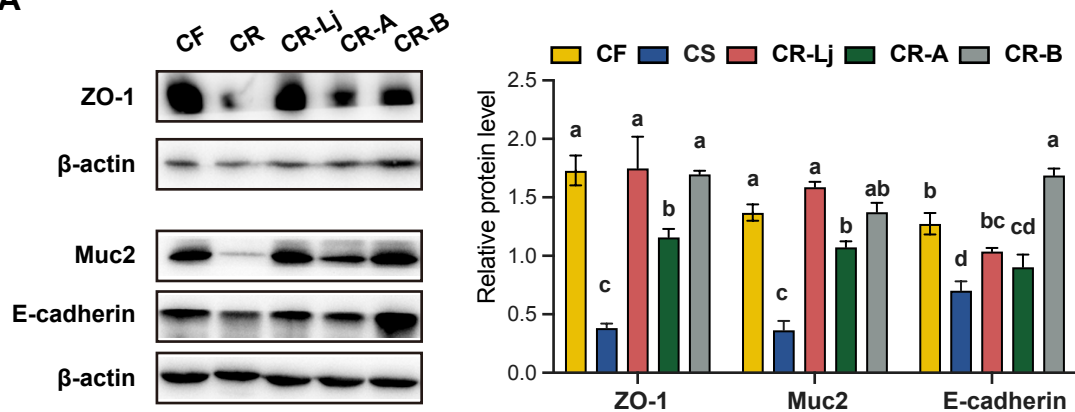**B**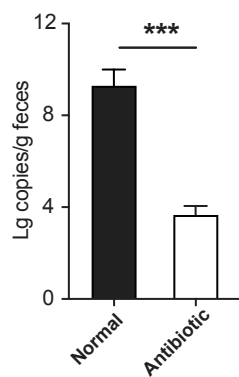**C**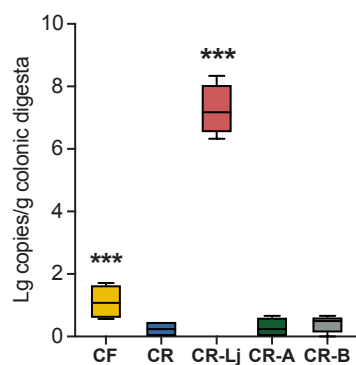**D**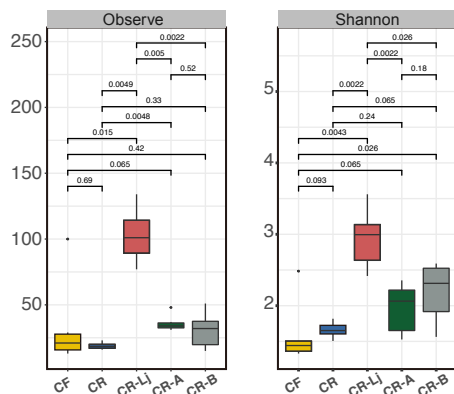**E**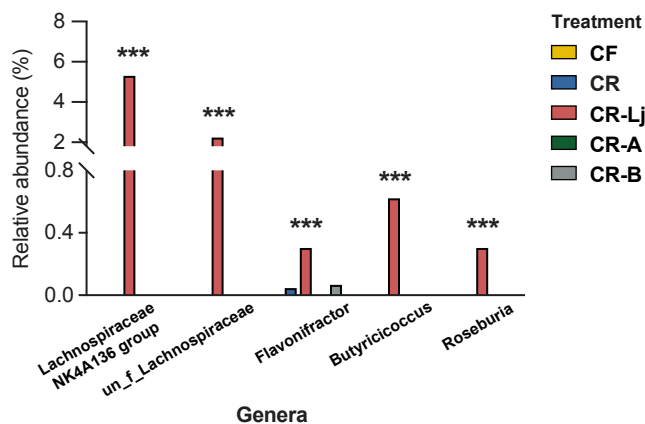

Supplement: Supplementary file 11 — Additional file 10: Figure S8. Microbial community following acetate, and butyrate intervention, related to Figs. 5 and 6. A Western blot of ZO-1, Muc2, and E-cadherin in the colon. B Copy number of fecal total bacteria in mouse on d 14. n = 8 ABX mice/group. C Copy number of L. johnsonii in the colon on d 28. n = 8 ABX mice/group. D Alpha diversity as measured by Observed species and Shannon diversity index in the colonic microbiome. E The relative abundance of genera with butyrate-conversion ability. CF, Fecal bacterial suspension of pigs in copper-free group. CR, Fecal bacterial suspension of pigs in copper-rich group. CR-Lj, Fecal bacterial suspension of pigs in copper-rich group with L. johnsonii. CR-A, Fecal bacterial suspension of pigs in copper-rich group with acetate. CR-B, Fecal bacterial suspension of pigs in copper-rich group with butyrate. Statistical analyses were performed using Kruskal–Wallis test with P value adjustment using FDR correction. One-way ANOVA with Tukey’s test, CR group versus other groups. ***p < 0.001. [file 40168_2023_1655_MOESM10_ESM.pdf]
